# Pathogen-specific stomatal responses in cacao leaves to *Phytophthora megakarya* and *Rhizoctonia solani*

**DOI:** 10.1038/s41598-025-94859-5

**Published:** 2025-03-27

**Authors:** Insuck Baek, Seunghyun Lim, Jae Hee Jang, Seok Min Hong, Louis K. Prom, Silvas Kirubakaran, Stephen P. Cohen, Dilip Lakshman, Moon S. Kim, Lyndel W. Meinhardt, Sunchung Park, Ezekiel Ahn

**Affiliations:** 1 Environmental Microbial and Food Safety Laboratory, Agricultural Research Service, United States, Department of Agriculture, Beltsville, MD 20705 USA; 2 Sustainable Perennial Crops Laboratory, Agricultural Research Service, United States, Department of Agriculture, Beltsville, MD 20705 USA; 3 Department of Civil Urban Earth and Environmental Engineering, Ulsan National Institute of Science and Technology, UNIST-gil 50, Ulsan, 44919 Republic of Korea; 4 Insect Control and Cotton Disease Research, Agricultural Research Service, Southern Plains Agricultural Research Center, United States, Department of Agriculture, College Station, TX 77845 USA; 5 Grape Genetics Research Unit, Agricultural Research Service, United States, Department of Agriculture, Geneva, NY 14456 USA; 6 Molecular Plant Pathology Laboratory, Agricultural Research Service, United States, Department of Agriculture, Beltsville, MD 20705 USA

**Keywords:** Cacao, Black pod rot, Stomatal response, Machine learning, Light conditions, Light responses, Plant stress responses, Stomata

## Abstract

Cacao is a globally significant crop, but its production is severely threatened by diseases, particularly Black Pod Rot (BPR) caused by *Phytophthora* spp. Understanding plant-pathogen interactions, especially stomatal responses, is crucial for disease management. Machine learning offers a powerful, yet largely untapped, approach to analyze and interpret complex plant responses in plant biology and pathology, particularly in the context of plant-pathogen interactions. This study explores the use of machine learning to analyze and interpret complex stomatal responses in cacao leaves during pathogen interactions. We investigated the impact of the black pod rot pathogen (*Phytophthora megakarya*) and a non-pathogenic fungus (*Rhizoctonia solani*) on stomatal aperture in two cacao genotypes (SCA6 and Pound7) under varying light conditions. Image analysis revealed diverse stomatal responses, including no change, opening, and closure, that were influenced by the interplay of genotype, pathogen isolate, and light conditions. Notably, SCA6 exhibited stomatal opening in response to *P. megakarya* specifically under a 12-hour light/dark cycle, suggesting a light-dependent activation of pathogen virulence factors. In contrast, Pound7 displayed stomatal closure in response to both *P. megakarya* and *R. solani*, indicating the potential recognition of conserved Pathogen-Associated Molecular Patterns (PAMPs) and a broader defense response. To further analyze these interactions, we employed machine learning techniques to predict stomatal area size. Our analysis identified key morphological features, with size-related traits being the strongest predictors. Shape-related traits also played a significant role when size-related traits were excluded from the prediction. This study demonstrates the power of combining image analysis and machine learning for discerning subtle, multivariate traits in stomatal dynamics during plant-pathogen interactions, paving the way for future applications in high-throughput disease phenotyping and the development of resistant crop varieties.

**Supplementary Information:**

The online version contains supplementary material available at 10.1038/s41598-025-94859-5.

## Introduction

Originating from Central and South America, the cacao plant (*Theobroma cacao* L.) has a history intertwined with human civilization dating back to at least 600 BC, when it was cultivated by the Pre-Columbian Maya people^1^. Today, it underpins the multi-billion dollar chocolate industry and provides livelihoods for millions of smallholder farmers worldwide^2^. Its uses extend beyond food, encompassing pharmaceuticals and cosmetics^2^. Cacao’s richness in polyphenols, particularly flavonoids, has drawn significant attention to the plant’s potential ability to alleviate certain health issues, including obesity and cancer^2^. Lamentably, the cacao plant suffers from the destructive impact of several diseases, such as black pod rot (BPR) caused by *Phytophthora* spp. ^3^. In response, there has been an effort to curate germplasm collections that possess resistance to those diseases. SCA6 and Pound7 are two genotypes of wild cacao from the Upper Amazon that display this much sought-after trait^3,4^.

Stomata are microscopic pores on leaf surfaces that play a pivotal role in plant-pathogen interactions. As most plant bacterial pathogens breach the epidermal barrier and exploit stomata for entry into the leaf apoplast^5^, plants have evolved a defense mechanism known as stomatal immunity. This mechanism triggers rapid stomatal closure upon pathogen detection, often within half an hour, to thwart bacterial invasion^5^. Such stomatal closure can be triggered by the plant’s detection of PAMPs, molecules uniquely conserved in both pathogenic and non-pathogenic microbes^6^. These telltale molecular signs of invasion are detected using Pattern Recognition Receptors (PRRs), which go on to trigger a series of intracellular reactions that lead to stomatal closure^6^. The reopening of stomata after bacteria-triggered closure is essential for plant health because it facilitates gas exchange, photosynthesis, and defense against a range of threats, including pathogens and herbivorous insects^7,8^. While plant-bacterial pathogen interactions and their influence on stomatal dynamics have been extensively studied, our understanding of plant-fungal or plant-oomycete interactions, particularly in cacao, remains limited. The diversity of stomatal responses to these pathogen types, including potential manipulations by pathogens to open stomata, is an area requiring further investigation.

Existing research on the oomycete pathogen *Phytophthora infestans* in potato (*Solanum tuberosum* L.) leaves revealed a pattern of stomatal opening at infection sites around 8 h post-inoculation (hpi), reaching maximum aperture at 24 h and remaining open thereafter for the development and release of spores^9^. *P. infestans* was found to release apoplastic factors that may lead to the opening of stomata, but further studies are needed to confirm the specific molecular interactions that take place^9^.

**Fig. 1:**
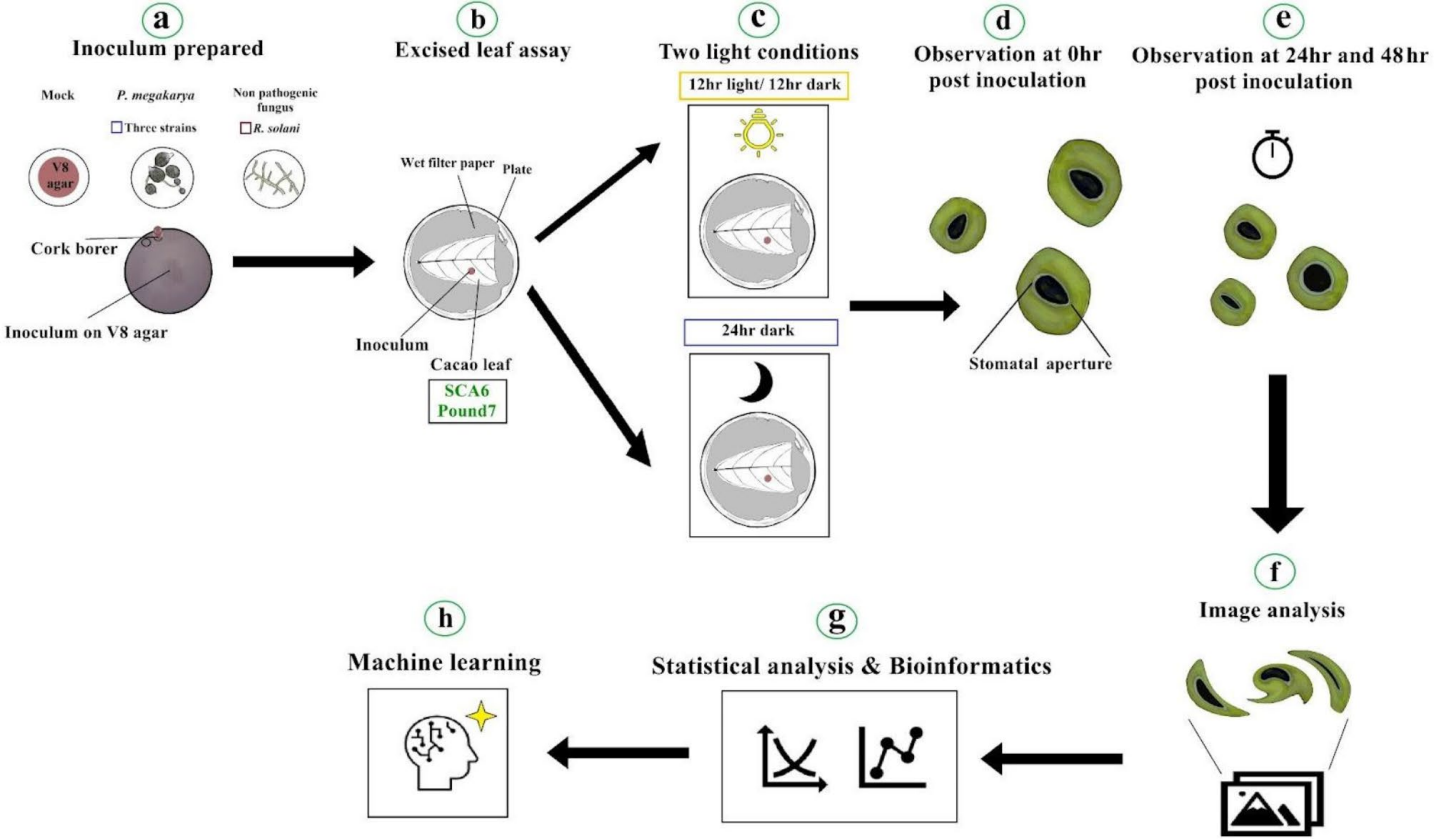
Research workflow and methodology. (**a**) Pathogen and mock inoculum (control) preparation. (**b**) Inoculation of excised cacao leaves (two genotypes). (**c**) Application of light (L) and dark (D) Conditions. (**d**, **e**) Time-series stomatal image acquisition (0, 24, 48 hpi). (**f**) Stomatal morphology image analysis. (**g**) Statistical and bioinformatics data analysis. (**h**) Machine learning for interaction analysis.

Intrigued by these findings and the knowledge gap in cacao-pathogen interactions, we designed a study to investigate stomatal aperture changes in cacao genotypes SCA6 and Pound7 when challenged by the oomycete pathogen *Phytophthora megakarya* Brasier & Griffin and the non-pathogenic fungus *Rhizoctonia solani* J.G. Kühn, 1858 under varying light conditions (Fig. 1). Our primary hypothesis was that stomatal responses would be influenced by complex interactions of cacao genotype, pathogen isolate, and light condition. Figure 1 provides a visual overview of our research workflow, outlining the steps from inoculum preparation to machine learning analysis. We cultured different isolates of *P. megakarya* and *R. solani* on agar plates and inoculated excised cacao leaves from two genotypes. These leaves were then subjected to different light conditions, and stomatal images were captured at various time points. Image analysis and advanced machine learning techniques were employed to analyze stomatal aperture dynamics and predict responses based on multiple variables. A key novelty of this work lies in the application of machine learning for pathogen classification based on stomatal responses, a largely unexplored area in plant pathology. Furthermore, our investigation into the interactions of *R. solani* and *P. megakarya* with specific cacao genotypes provides valuable, novel insights into cacao-pathogen interactions. Given the multivariate nature of stomatal responses in our study, machine learning methodology is uniquely suited for this analysis. Unlike conventional statistics, machine learning enables robust predictive modeling of stomatal behavior in response to the complex interplay of genotype, pathogen, and environment, and facilitates the discovery of key morphological features driving these responses. Our findings confirmed the intricate relationship between plant genotype, pathogen isolate, light condition, and time, which significantly impacted stomatal aperture dynamics.

We leveraged nine different machine learning algorithms, including Bootstrap Forest (a type of Random Forest), Support Vector Machines (SVM), and Neural Networks, to predict stomatal aperture size based on a combination of morphological features, genotype, pathogen isolate, and environmental conditions. This approach allowed us to not only assess the predictive power of different models but also to identify the key features driving changes in stomatal aperture. This pioneering application of machine learning in cacao research sheds light on the multifaceted nature of stomatal aperture regulation and paves the way for future investigations into stomatal-pathogen-environment interactions in other crops. Beyond advancing our understanding of cacao-pathogen interactions, our findings have direct implications for cacao breeding programs. Identifying specific stomatal traits associated with resistance to *P. megakarya* and *R. solani*, as enabled by our machine learning approach, can inform targeted breeding strategies. Breeders could utilize these identified traits as selection criteria to develop new cacao varieties with enhanced and durable resistance to economically important diseases like Black Pod Rot. Furthermore, the ability to distinguish between pathogen treatments based on stomatal morphology suggests the potential for developing rapid, image-based diagnostic tools to identify not only the presence of disease, but also potentially the specific fungal isolate or species involved. This could enable more targeted and effective disease management strategies, contributing to improved cacao yields and farmer livelihoods. The insights gleaned from this study hold promise for the development of innovative disease management strategies in agriculture.

## Results

### Stomatal responses to control, *P. megakarya* ZTH0145, and *P. megakarya* GH8

**Fig. 2:**
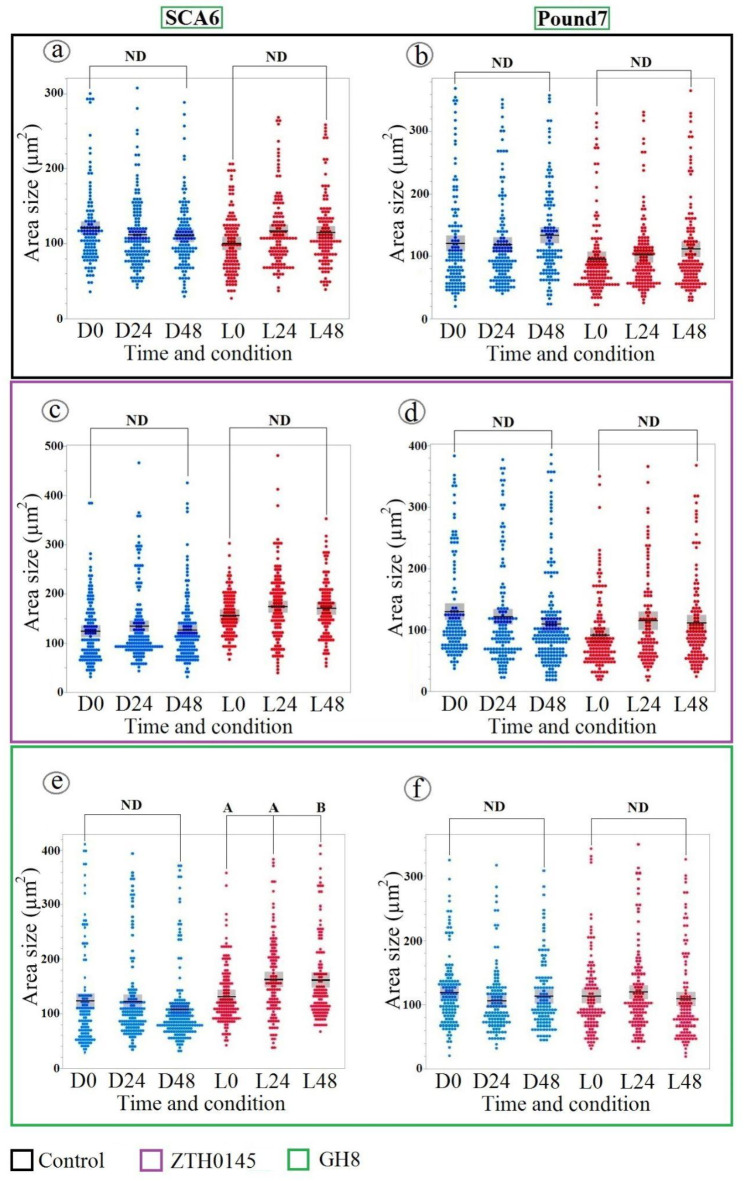
Stomatal aperture dynamics in cacao genotypes under different inoculation and light conditions. Stomatal aperture dynamics in SCA6 (**a**, **c**, **e**) and Pound7 (**b**, **d**, **f**) cacao genotypes under different inoculation and light conditions. Scatterplots illustrate stomatal aperture size (µm²) under D (blue) and L (12-hour light/12-hour dark, red) conditions over time (0, 24, and 48 h). (**a**, **b**): Control. (**c**, **d**): *P. megakarya* ZTH0145 inoculation. (**e**, **f**): *P. megakarya* GH8 inoculation. Solid horizontal lines represent mean values, with boxes indicating confidence intervals. No significant differences in stomatal aperture were observed for control and *P. megakarya* ZTH0145 inoculation across genotypes, light conditions, or time points (ANOVA, *p* > 0.05). Stomatal aperture significantly increased in SCA6 under the L following GH8 inoculation (Tukey’s HSD, *p* < 0.03), while remaining stable in SCA6 under D conditions and in Pound7 under both conditions. Different alphabetical letters indicate statistically significant differences between group means, as determined by Tukey’s HSD test. ND indicates no significant difference between groups connected by brackets, as determined by Tukey’s HSD test (*p* > 0.05).

To assess the baseline stomatal dynamics and the impact of specific *P. megakarya* isolates, we examined stomatal aperture changes in response to control, inoculation with *P. megakarya* isolate ZTH0145, and inoculation with *P. megakarya* isolate GH8 under both L and D conditions (Fig. 2). We observed no significant changes in stomatal aperture in either SCA6 or Pound7 following control or inoculation with *P. megakarya* isolate ZTH0145. This indicates that, within the experimental timeframe, these treatments did not induce detectable changes in stomatal behavior. However, inoculation with *P. megakarya* isolate GH8 led to a significant increase in stomatal aperture. This increase was observed at 48 hpi with isolate GH8 and was statistically significant according to Tukey’s HSD test (*p* < 0.03). In contrast, Pound7 showed no significant change in stomatal aperture under either light condition, suggesting the influence of both cacao genotype and environmental lighting on stomatal responses.

### Stomatal responses to *P. megakarya* isolate GH21 infection in cacao genotypes SCA6 and Pound7

**Fig. 3:**
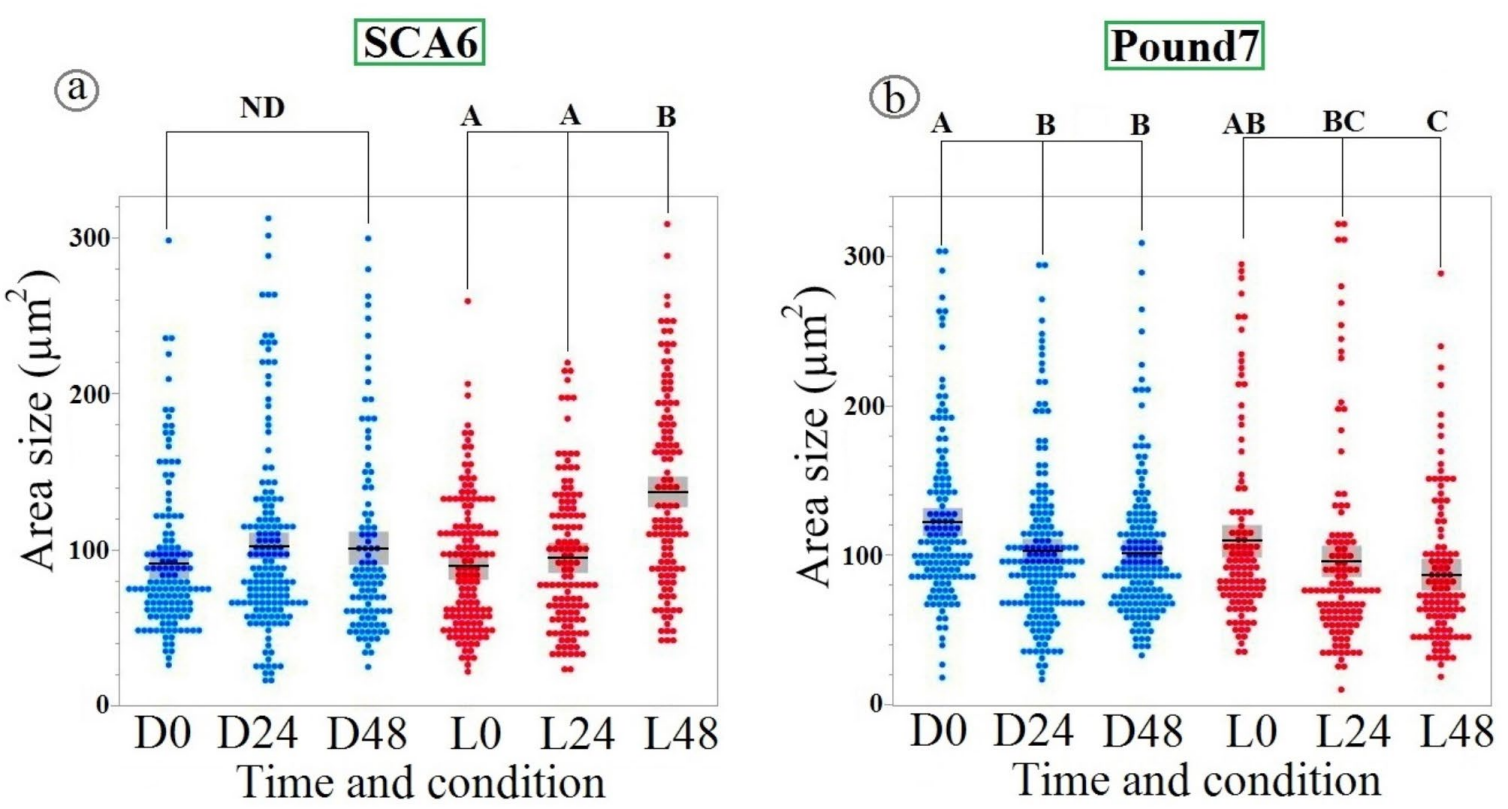
Differential stomatal responses to *P. megakarya* isolate GH21 in cacao genotypes. Scatterplots depict stomatal aperture size (µm²) in SCA6 (**a**) and Pound7 (**b**) genotypes under D (blue) and L (red) conditions at 0, 24, and 48 h post-inoculation. Mean values are represented by horizontal lines, with confidence intervals indicated by boxes. A significant increase in stomatal aperture was observed in SCA6 under L condition at 48 h post-inoculation with GH21 (Tukey’s HSD, *p* < 0.0001). In contrast, stomatal aperture decreased in Pound7 under both L and D conditions (*p* < 0.05). Different alphabetical letters indicate statistically significant differences between group means (Tukey’s HSD). ND indicates no significant difference between groups connected by brackets, as determined by Tukey’s HSD test (*p* > 0.05).

Building upon the observed responses to isolate GH8, *P. megakarya* isolate GH21 further emphasizes the genotype-specific stomatal dynamics (Fig. 3). GH21 also triggered light-dependent stomatal opening in SCA6. However, in a notable divergence from GH8, GH21 elicited stomatal closure in Pound7. This closure, unlike the SCA6 response, was observed consistently under both light and dark conditions. This contrasting pattern, light-dependent opening in SCA6 versus light-independent closure in Pound7 in response to GH21, reinforces the emerging picture of distinct stomatal regulatory mechanisms operating in these cacao genotypes, and further underscores the isolate-specific nature of *P. megakarya*-cacao interactions.

### Stomatal responses to *R. solani* isolate GC33-C inoculation in cacao genotypes SCA6 and Pound7

**Fig. 4:**
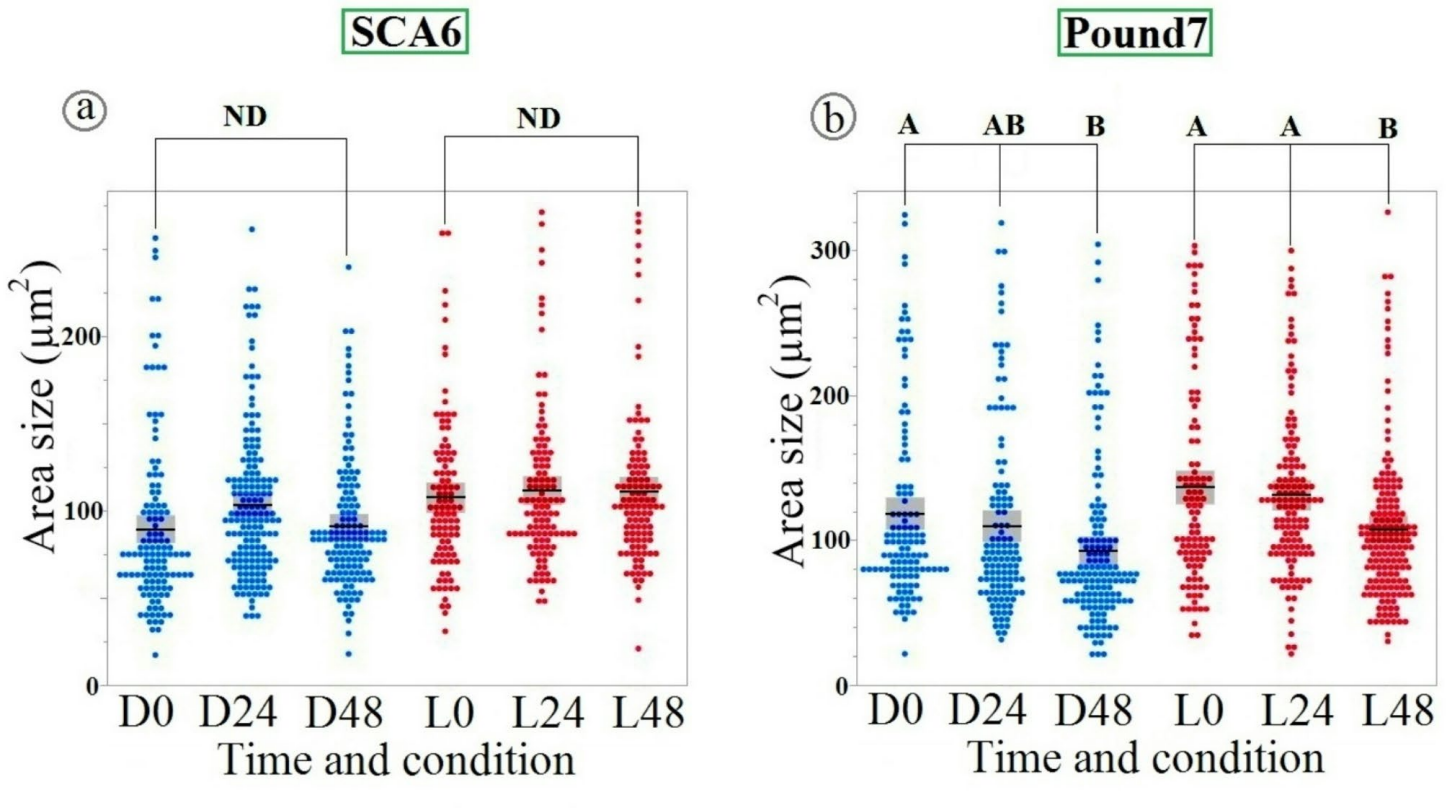
Contrasting stomatal responses to *R. solani*. Isolate GC33-C in Cacao Genotypes. Scatterplots illustrate stomatal aperture size (µm²) in SCA6 (**a**) and Pound7 (**b**) genotypes under D (blue) and L (red) conditions at 0, 24, and 48 h post-inoculation. Mean values are represented by horizontal lines, with confidence intervals indicated by boxes. While stomatal aperture remained stable in SCA6, a significant decrease was observed in Pound7 under both L and D conditions at 48 h post-inoculation. Different alphabetical letters indicate statistically significant differences (Tukey’s HSD). ND indicates no significant difference between groups connected by brackets, as determined by Tukey’s HSD test (*p* > 0.05).

*R. solani* inoculation (Fig. 4) revealed a fundamental difference in stomatal response patterns between cacao genotypes. SCA6 demonstrated a consistently narrow response range, remaining unresponsive to *R. solani*, similar to its lack of response to control and *P. megakarya* ZTH0145. In contrast, Pound7 exhibited a broadly responsive stomatal closure, triggered not only by pathogenic *P. megakarya*, but also by the non-cacao pathogen *R. solani*, and, importantly, under both L and D conditions. This broad responsiveness of Pound7, especially its stomatal closure to the non-pathogenic *R. solani*, further substantiates the hypothesis of a more generalized defense mechanism in Pound7 compared to the more specific pathogen recognition in SCA6.

### Impact of pathogen inoculation on stomatal circularity in Cacao genotypes under continuous dark

**Fig. 5:**
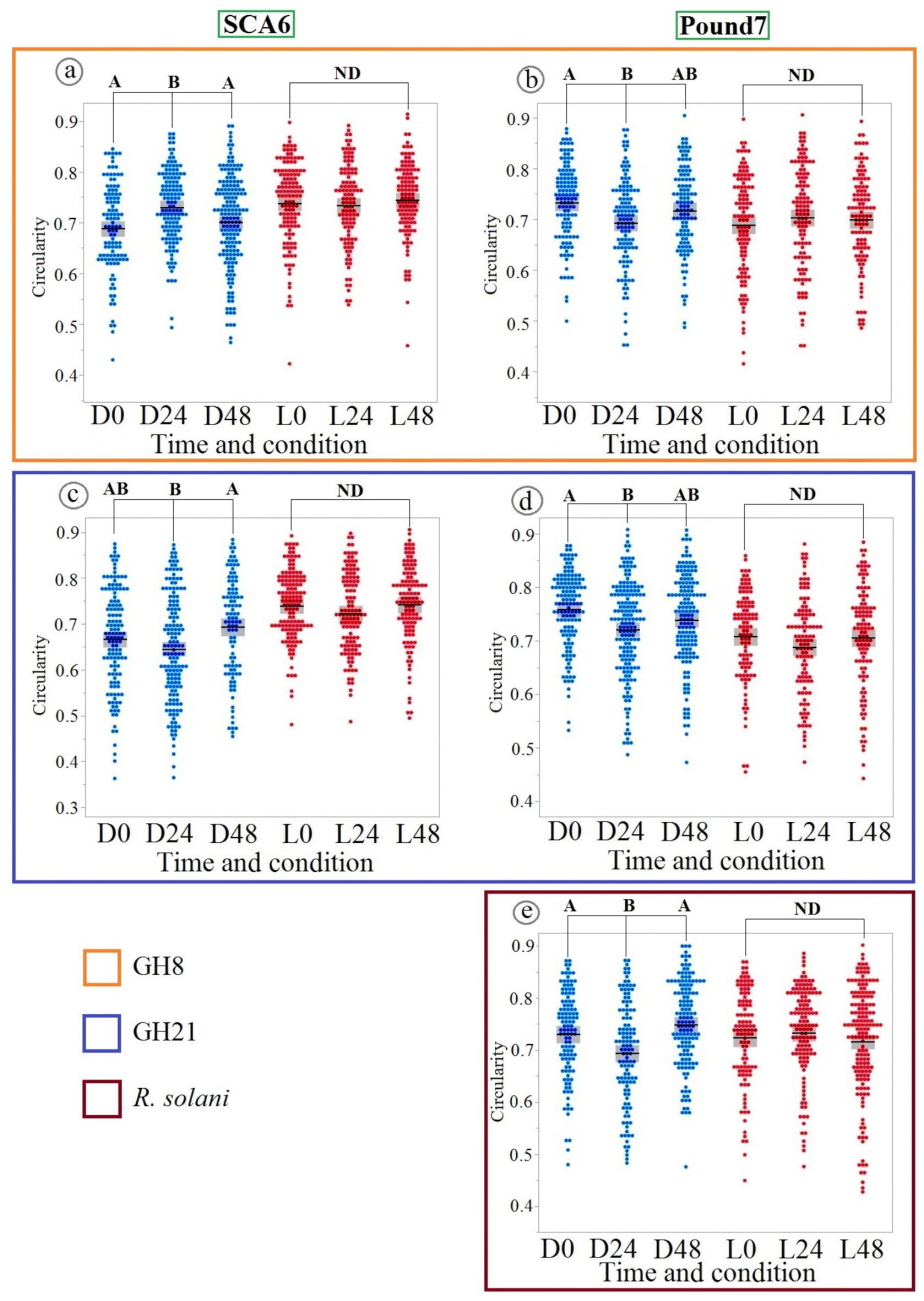
Changes in stomatal circularity under dark conditions in cacao genotypes following pathogen inoculation. (**a**) In the SCA6 genotype inoculated with GH8, a significant increase in circularity was observed at 24 h under D conditions. (**b**) In Pound7 inoculated with GH8, there was a statistically significant decrease in circularity at 24 h under D conditions. (**c**) When SCA6 was inoculated with GH21, circularity was significantly lower at 24 h under D compared to 48 h under D. (**d**) In Pound7 inoculated with GH21, circularity decreased at 24 h under D compared to 0 h. (**e**) Pound7 inoculated with *R. solani*. showed significantly lower circularity at 24 h under D than other time points. Different letters above data points indicate statistically significant differences between time points (Tukey’s HSD test). ND indicates no significant difference between groups connected by brackets, as determined by Tukey’s HSD test (*p* > 0.05).

To further investigate stomatal morphology beyond aperture area, we analyzed stomatal circularity, a key indicator of shape alterations (Fig. 5). This analysis revealed that pathogen-induced changes in stomatal shape, unlike aperture area responses, were strikingly specific to D conditions and genotype. Specifically, both SCA6 and Pound7 exhibited significant alterations in stomatal circularity at 24 hpi exclusively under D conditions, and only in response to inoculation with the pathogenic *P. megakarya* isolates GH8 and GH21. Intriguingly, Pound7 also displayed significant circularity changes upon inoculation with the non-cacao pathogen *R. solani* under dark conditions. Notably, neither control nor inoculation with ZTH0145, regardless of light condition, induced any detectable changes in stomatal circularity, suggesting that these shape changes are elicited by a distinct and limited set of pathogen-genotype-environment interactions.

### Multivariate analysis of stomatal traits

**Fig. 6:**
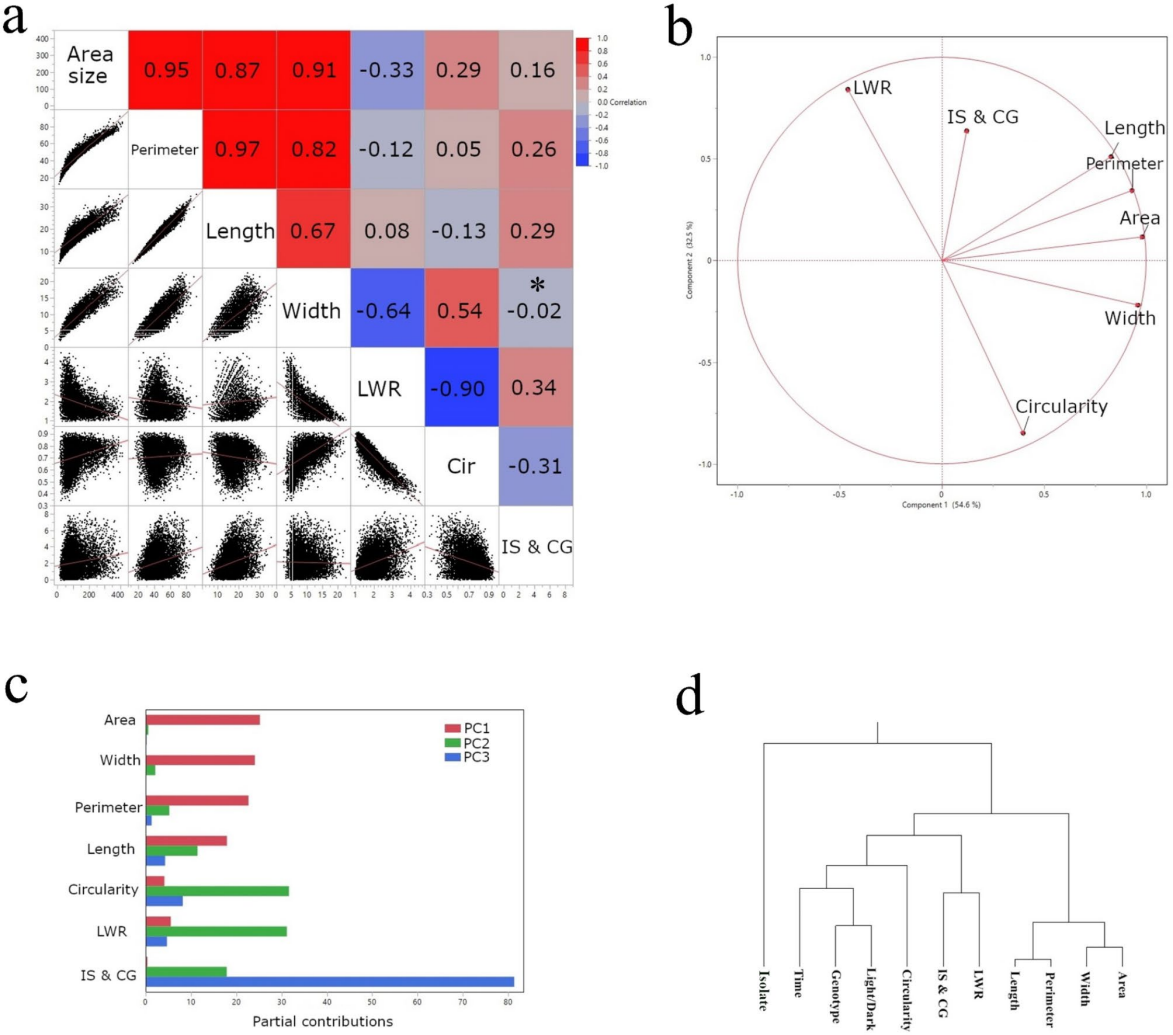
Relationships among stomatal traits. (**a**) Pearson correlation coefficients between pairs of stomatal traits. Red indicates positive correlations, blue indicates negative correlations, and color intensity reflects the strength of the correlation. * indicates insignificance with a *p*-value of 0.0632. (**b**) PCA biplot showing the relationships between stomatal traits and the first two principal components (PC1 and PC2). The direction and length of the vectors indicate how each trait contributes to the principal components. (**c**) Contribution of individual stomatal traits to the first three principal components. (**d**) Hierarchical clustering analysis of stomatal traits using Ward’s linkage method.

To explore the relationships among stomatal traits, we performed a multivariate analysis encompassing Pearson correlation, principal component analysis (PCA), and hierarchical clustering for traits. Figure 6a presents a correlation matrix summarizing the relationships between pairs of the following stomatal traits: area size, perimeter, length, width, length-to-width ratio (LWR), circularity, and the intersection of length and width (IS) and center of gravity (CG). Strong positive correlations were observed between area size and other size-related traits such as perimeter (*r* = 0.97, *p* < 0.0001), length (*r* = 0.67, *p* < 0.0001), and width (*r* = -0.64, *p* < 0.0001). Except for the width-the distance between the intersection of length & width (IS) and center of gravity (CG) (IS & CG) pair, all pairs showed statistical significance (all *p* < 0.0001), showing a strong correlation between stomatal size and shape. PCA was conducted to further visualize the interrelationships among traits and identify key combinations of traits that explain the variation in stomatal morphology (Fig. 6b). The first two principal components (PC1 and PC2) accounted for 87.1% of the total variance. The partial contribution of variables (Fig. 6c) revealed that area size, width, perimeter, and length were nearly identical based on PC1 through 3, while circularity and LWR were very similar to each other. The distance between the IS & CG was distinct, with a high portion of PC3. Hierarchical clustering analysis (Fig. 6d) revealed distinct groupings among the traits. Size-related traits (area, perimeter, length, and width) clustered together, while circularity and LWR formed a separate cluster. LWR and IS & CG were grouped together, while light condition and genotype were grouped together. The pathogen isolate treatment was apart from the rest of the traits.

### Machine learning analysis

**Fig. 7:**
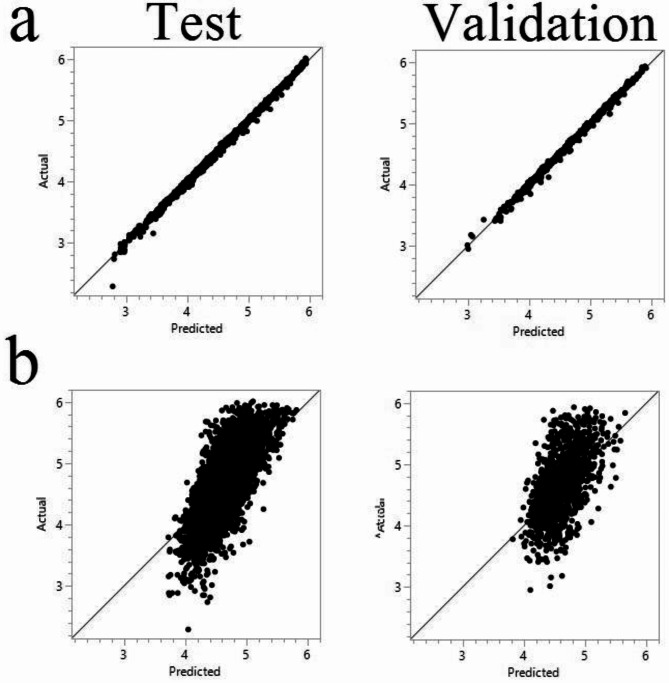
Scatter plots comparing actual versus predicted stomatal area size values. (**a**) Model performance on the test set when all stomatal traits, genotype, pathogen isolate, and light condition are included as predictors. The close alignment of points to the diagonal indicates high predictive accuracy (R-square for training set = 0.999, validation set = 0.998). (**b**) Model performance on the test set when the three traits directly associated with stomatal area (perimeter, length, and width) are excluded as predictors. There is an increase in variability compared to (**a**) (R-square for training set = 0.672, validation set = 0.302). The diagonal line represents perfect prediction.

We employed machine learning techniques to predict stomatal area size to further investigate the factors influencing stomatal aperture. We evaluated the performance of nine different models (Tables 1 and 2): Boosted Tree^10^, Bootstrap Forest^11^, Decision Tree^12^, Least Squares^13^, Stepwise^14^, Generalized Regression Lasso^15^, K-Nearest Neighbors (k-NN)^16^, Neural Boosted^17^, and SVM-RBF model^18^. When all stomatal size and shape-related traits, genotype, pathogen isolate, and light condition were included as predictors (Table 1; Fig. 7a), all models demonstrated exceptional predictive accuracy, with R-squared values exceeding 0.90 on both the training and validation sets. However, when excluding the three traits directly associated with stomatal area (perimeter, length, and width), all models experienced a dramatic decrease in predictive accuracy (Table 2; Fig. 7b). Bootstrap Forest and Boosted Tree remained the top-performing models, followed by SVM-RBF and Neural Boosted, while k-NN performed poorly. This could be attributed to k-NN’s sensitivity to irrelevant features and its struggle to handle high-dimensional data, especially when key features are removed. Analysis of feature importance in the Bootstrap Forest model (Table 3) revealed that when all traits were included, perimeter and width were the most influential predictors of stomatal area size, explaining 70.04% and 20.25% of the model’s predictive power, respectively. This displays the importance of these direct size-related features in determining stomatal aperture. When excluding the direct area-related traits, stomatal shape-related traits such as LWR, IS & CG, and circularity emerged as the most important predictors (sum = 76.22%), followed by light conditions (3.98%) and genotypes (3.66%). Among pathogen treatments, isolate GH21 had the most influence on the model (2.59%), followed by ZTH0145 (2.48%), while the control condition (mock inoculation) and *R. solani* inoculation had no impact (0%).

**Table 1:** Performance of machine learning models in predicting stomatal area size using all available traits.

Training set		Validation set	
Method	R-square	Method	R-square
Stepwise	1	Stepwise	1
Least Squares	1	Least Squares	1
Neural Boosted	1	Neural Boosted	1
Generalized Regression Lasso	1	Generalized Regression Lasso	1
Boosted Tree	0.9996	Boosted Tree	0.9989
Bootstrap Forest	0.9989	Bootstrap Forest	0.9976
Decision Tree	0.9975	Decision Tree	0.9952
SVM-RBF	0.9967	SVM-RBF	0.9951
k-NN	0.9362	k-NN	0.9355

This table compares the accuracy of different machine learning models in predicting stomatal area size when all available traits are used. R-square values indicate the proportion of variance in stomatal area size explained by the model, with higher values indicating better predictive performance. As shown above, all nine machine learning algorithms achieved nearly perfect accuracy for both training and validation sets.

**Table 2:** Predictive performance of machine learning models using indirect stomatal traits.

Training set		Validation set	
Method	R-square	Method	R-square
Bootstrap Forest	0.6799	Boosted Tree	0.3066
Boosted Tree	0.5089	Bootstrap Forest	0.2875
SVM-RBF	0.3346	Neural Boosted	0.2852
Neural Boosted	0.300	SVM-RBF	0.2846
Decision Tree	0.2730	Decision Tree	0.2351
Stepwise	0.2031	k-NN	0.2146
Least Squares	0.2031	Least Squares	0.2043
Generalized Regression Lasso	0.2031	Stepwise	0.2043
k-NN	0.1952	Generalized Regression Lasso	0.2042

This table compares the accuracy of different machine learning models in predicting stomatal area size when excluding traits directly related to area (perimeter, length, and width). Both training and validation sets demonstrate drastically lower accuracy compared to those shown in Table 1.

**Table 3:** Feature importance in the bootstrap forest model for predicting stomatal area size.

Traits	Number of splits	Sum of squares	Importance	Portion
Feature importance scores from bootstrap forest: all predictors				
Perimeter	21,003	584.41834	18	0.7004
Width	5394	168.938444	5	0.2025
Length	4491	58.092242	1	0.0696
Circularity	22,550	19.4323569	0	0.0233
LWR	3073	2.76664021	0	0.0033
IS & CG	3893	0.29570011	0	0.0004
Light condition	2403	0.07045057	0	0.0001
Genotype	2189	0.06636906	0	0.0001
Time-24 h	1735	0.06047857	0	0.0001
Time-48 h	1714	0.05170482	0	0.0001
Time-0 h	1689	0.05034324	0	0.0001
Control	976	0.03697445	0	0
GH21	927	0.03433291	0	0
ZTH0145	874	0.03239024	0	0
GH8	880	0.02685344	0	0
*R. solani*	947	0.02588579	0	0
Feature importance scores from bootstrap forest: indirect predictors only				
LWR	4674	169.498324	18	0.3037
IS & CG	4899	154.556283	16	0.2769
Circularity	4433	101.337893	10	0.1816
Light condition	1679	22.1868253	2	0.0398
Genotype	972	20.4144647	2	0.0366
GH21	674	14.475029	1	0.0259
ZTH0145	862	13.8166625	1	0.0248
Time-0 h	965	12.2695852	1	0.022
Time-24 h	1045	11.0383978	1	0.0198
Time-48 h	1001	10.9940128	1	0.0197
GH8	895	9.44258476	1	0.0169
*R. solani*	844	9.11265927	0	0.0163
Control	856	8.9655562	0	0.0161

This table presents the ranking of feature importance in the Bootstrap Forest model for predicting stomatal area size. The top panel includes all traits, while the bottom panel excludes traits directly related to area (perimeter, length, and width). Importance is measured by the number of splits (representing how often a feather was used to divide the data) and the sum of squares (reflecting the improvement in model accuracy due to a feature), with higher values indicating greater influence on the model’s predictions. Portion presents the relative contribution of each feature to the overall constructed model.

**Fig. 8:**
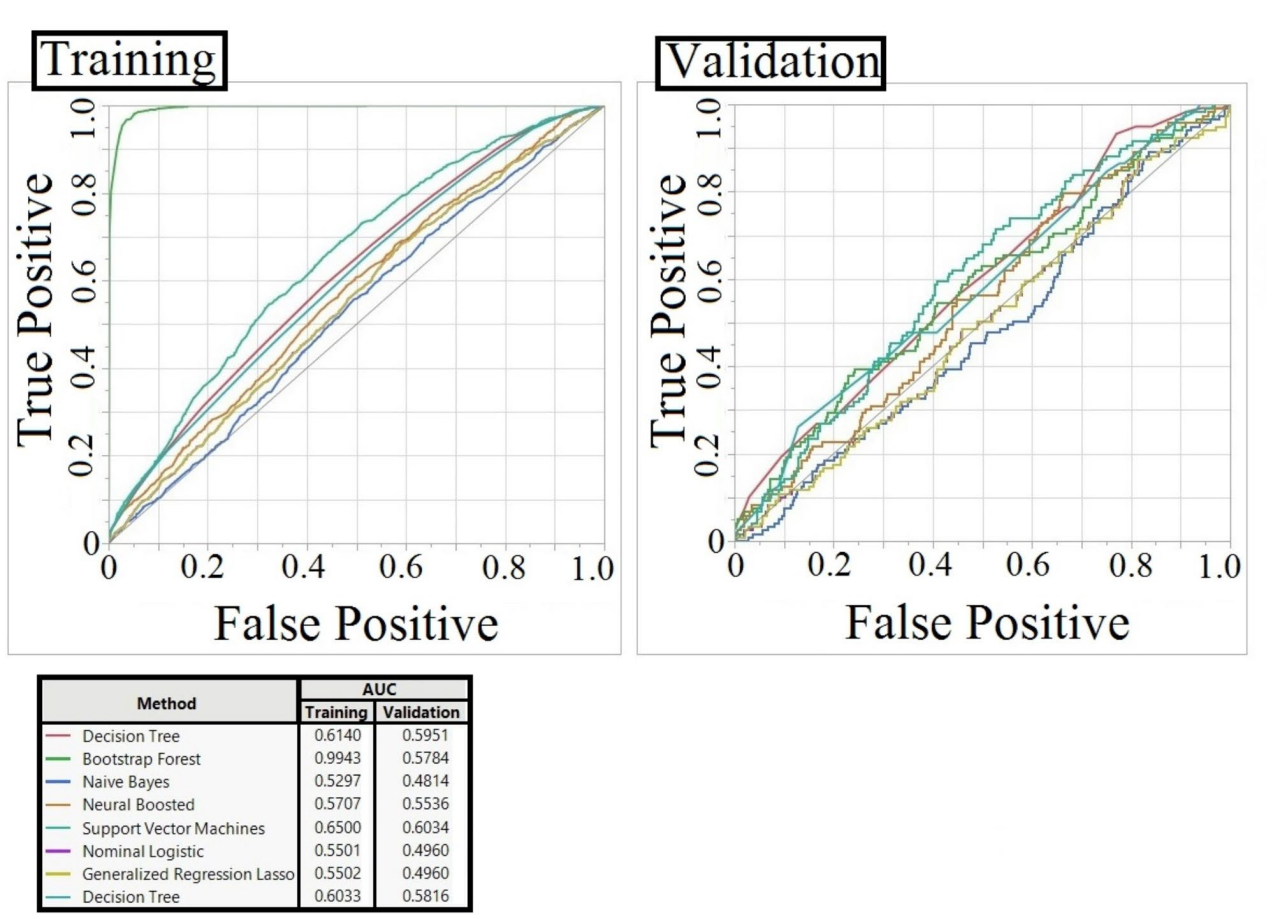
Receiver operating characteristic (ROC) curves and area under the curve (AUC) values for eight machine learning models in classifying pathogen treatments. The ROC curves illustrate the trade-off between true positive rate (sensitivity) and false positive rate (1-specificity) for each model. Higher AUC values indicate better classification performance. The models were trained on 80% of the data and validated on the remaining 20%, using 10-fold cross-validation.

To assess the ability of machine learning to differentiate between pathogen treatments based on stomatal traits, we performed a classification analysis. Figure 8 shows the Receiver Operating Characteristic (ROC) curves and Area Under the Curve (AUC) values for eight different machine learning models. Most models achieved AUC values significantly higher than 0.2, which would be expected by random chance with five classes (control, *R. solani*, and three *P. megakarya* isolates). Decision Tree, Bootstrap Forest, Boosted Tree, and SVM-RBF models exhibited the highest classification performance, with AUC values nearly 0.6 for the validation set. Notably, the Bootstrap Forest model showed a higher AUC on the training set compared to the validation set, suggesting potential overfitting to the training data. In contrast, the SVM-RBF model maintained similar AUC values for both training and validation sets, indicating better generalization and capturing complex non-linear relationships.

To better understand the key features influencing pathogen treatment classification, we analyzed the feature importance scores derived from our SVM-RBF model (Table 4). Our analysis revealed that stomatal length was the most significant factor, followed by circularity and the IS & CG. These results suggest that the morphological characteristics of stomata, particularly stomatal length, could serve as strong indicators for classifying different pathogens, especially when supported by larger datasets in future studies.

**Table 4:** Feature importance scores for the SVM-RBF model in classifying pathogen treatments.

Trait	Main effect	Total effect	Importance
Length	0.09	0.37	
Circularity	0.076	0.314	
IS & CG	0.051	0.277	
Perimeter	0.058	0.242	
Width	0.048	0.201	
Area	0.036	0.188	
LWR	0.026	0.158	
Genotype	0.026	0.108	
Light condition	0.015	0.08	
Time-0 h	0.009	0.029	
Time-48 h	0.007	0.021	
Time-24 h	0.007	0.018	

The table shows the relative contribution of each feature to the model’s ability to distinguish between different pathogen treatments (control, *R. solani*, and three *P. megakarya* isolates). Higher values indicate greater importance in the classification task. “Main Effect” represents the direct effect of each feature, while “Total Effect” includes both the direct and indirect effects through interactions with other features.

## Discussion

Cacao, vital to the chocolate industry and countless livelihoods, faces the constant threat of pathogens. *Phytophthora* spp., the causal oomycete of BPR, is particularly devastating, leading to substantial pod losses and tree mortality^19^. Understanding plant-pathogen interactions, particularly stomatal responses, is crucial for developing effective disease management strategies. Our study revealed a complex interplay of cacao genotype, pathogen isolate, light conditions, and time in shaping stomatal dynamics.

Stomatal movement is influenced by environmental cues and circadian rhythms, playing a key role in plant-pathogen interactions^20^. Plants employ PAMP-triggered stomatal defense mechanism to close stomata upon pathogen detection. In Arabidopsis, this closure is also mediated by effector-triggered immunity upon recognition of a specific pathogen effector AvrRpt2^6,21,22^. However, successful pathogens have evolved virulence mechanisms to manipulate stomata, using toxins or effectors to force entry into the plant host^20^.

**Fig. 9:**
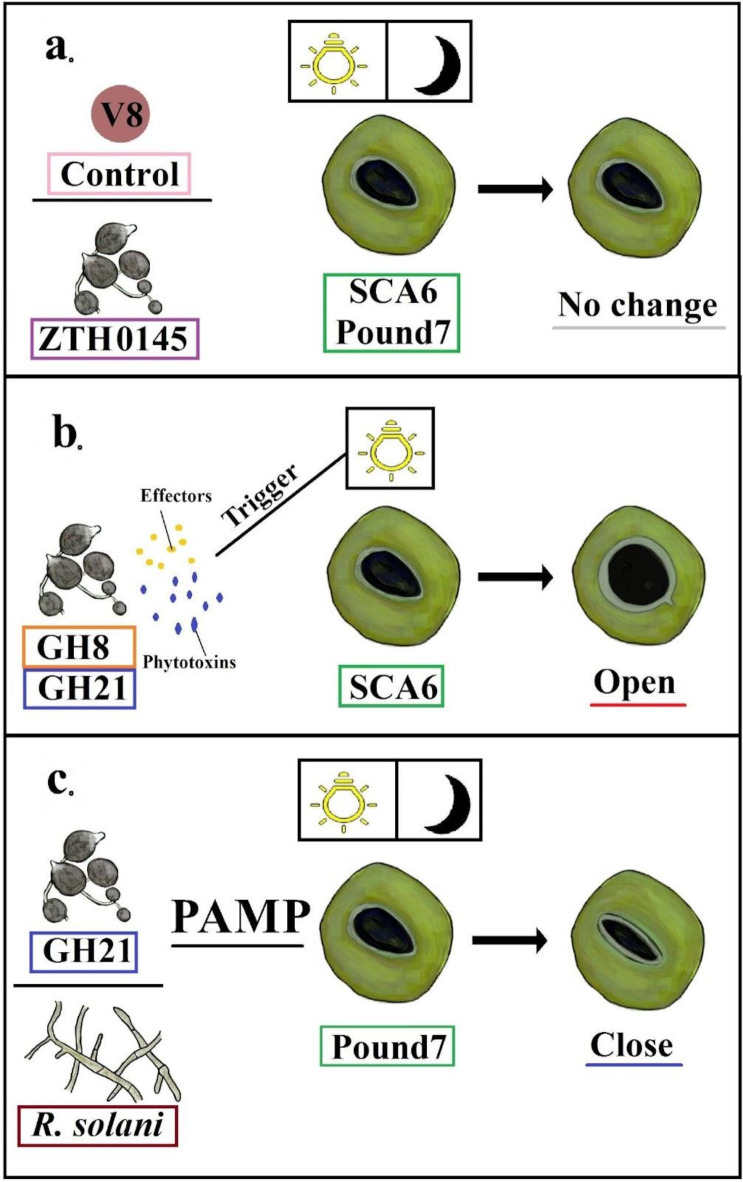
Hypothetical model of stomatal responses in cacao to different pathogens and light conditions. (**a**) No Response to Control and ZTH0145: Mock inoculation and inoculation with *P. megakarya* isolate ZTH0145 did not induce stomatal aperture changes in either SCA6 or Pound7 cacao genotypes under both light conditions. (**b**) Light-Dependent Stomatal Opening in SCA6: *P. megakarya* isolates GH8 and GH21 triggered stomatal opening in SCA6 only under the 12 h light/12 h dark cycle (L), suggesting a potential role of light in promoting the action of effectors or phytotoxins that induce stomatal opening. (**c**) PAMP-Triggered Stomatal Closure in Pound7: *P. megakarya* isolate GH21 and the non-pathogenic fungus *R. solani* induced stomatal closure in Pound7 under both light conditions, likely through the activation of PAMP-triggered immunity. The response to *R. solani* suggests a broad recognition mechanism in Pound7.

While previous research has primarily focused on bacterial pathogens and their interactions with stomata, our findings highlight cacao stomata’s distinct and varied responses to an oomycete pathogen and a non-pathogenic fungus, as summarized in Fig. 9.

Interestingly, both SCA6 and Pound7, despite being BPR-resistant genotypes^23^, exhibited different stomatal responses to *P. megakarya*. SCA6 showed light-dependent stomatal opening in response to specific isolates (GH8 and GH21), potentially indicating an attempt by the pathogen to manipulate stomatal aperture for successful invasion (Fig. 9b). This is consistent with previous studies suggesting that some pathogens can actively promote stomatal opening to facilitate their entry into the plant^24^. For instance, the toxin fusicoccin, produced by the fungus *Fusicoccum amygdali*, forces stomata open even in darkness^25^. Other pathogens, like *Sclerotinia sclerotiorum*, utilize oxalate to influence stomatal regulatio^26,27^. We also observed no significant changes in stomatal aperture in either SCA6 or Pound7 following control or inoculation with ZTH0145. One possible explanation for this differential response, specifically the lack of response to ZTH0145, is a quantitative difference in virulence factor production. ZTH0145 may produce stomata-modulating effectors or toxins at lower levels or with delayed kinetics compared to GH8 and GH21, rendering their effects undetectable within our 48-hour experimental timeframe. Therefore, ZTH0145 might still possess the capacity to influence stomata, but its effect may require longer incubation times or be below the threshold of detection within our experimental timeframe. Further investigation, perhaps involving time-course studies with longer observation periods or direct quantification of virulence factor production across isolates, would be necessary to validate this hypothesis.

Pound7 consistently displayed stomatal closure to both pathogenic and non-pathogenic microbes. This response potentially demonstrates a robust defense mechanism, prioritizing the prevention of pathogen entry (Fig. 9c). This response may be mediated by PAMP-triggered immunity (PTI), a basal defense response activated upon recognition of conserved PAMPs^28^. This involves the recognition of conserved microbial molecules by Pattern Recognition Receptors (PRRs) on guard cells, leading to downstream signaling events that ultimately affect ion channel activity and stomatal aperture. Key signaling components likely include calcium ions (Ca2+), reactive oxygen species (ROS), and potentially plant hormones such as abscisic acid (ABA)^29,30^. Notably, isolates GH8 and GH21, both isolated from Papanwie, Volta in Ghana, elicited similar responses in SCA6; meanwhile, ZTH0145, isolated from Kedia in Cameroon, did not. The differing responses to isolates from distinct geographical origins emphasize the impact of pathogen genetic diversity on the co-evolutionary arms race between host and pathogen^31^. This highlights the ongoing evolutionary struggle between cacao and *P. megakarya*, where pathogen populations adapt to overcome host defenses, and host plants evolve new resistance mechanisms. *R. solani* likely possesses PAMPs recognized by Pound7, triggering stomatal closure. This ‘first line of defense’ likely enhances resistance to a broad range of pathogens^9^, but may entail trade-offs. Constitutive closure can restrict CO2 uptake, limiting photosynthesis and potentially impacting growth^32^, and sustained defense activation can be energetically costly^33^. From an evolutionary perspective of cacao, SCA6 might favor a ‘tolerance’ strategy, accepting some infection to maintain growth, whereas Pound7 may prioritize ‘resistance’ via stomatal closure, even to non-pathogens. These contrasting approaches likely reflect different ecological pressures and fitness trade-offs.

On non-host plant species, rust pathogens often fail to locate and penetrate stomata accurately, preventing infection. This inability has been attributed to non-host plants emitting inappropriate biochemical or thigmotropic signals^24,34–36^. The interaction between a pathogen and a non-host plant can be complex and influenced by various factors. This concept of host-specificity also applies to pathogens like the fungus *R. solani*, which has diverse subgroups with varying host ranges, potentially linked to their ability to recognize and respond to specific host signals^37^. Some fungi and oomycetes also exploit stomata as an exit point for spore dispersal after successfully colonizing the leaf^24,38^, highlighting the multifaceted role of stomata in plant-pathogen interactions. Even in non-pathogenic interactions, plants can recognize and respond to fungi, potentially triggering defense mechanisms that also hinder the growth of true pathogens, further highlighting the complex and potentially beneficial interplay between plants and fungi^39^.

The observation that changes in stomatal circularity were predominantly associated with the dark condition and specific isolates (GH8 and GH21, but not ZTH0145) warrants further investigation. It is speculated that the observed changes in stomatal circularity under dark conditions result from a combination of pathogen-derived factors and altered plant cell physiology. Darkness likely modifies plant hormone signaling and cellular processes, potentially increasing guard cell sensitivity to pathogen effectors or toxins^40^ that influence cytoskeletal arrangements^41,42^ and turgor pressure^43^, ultimately affecting stomatal shape.

The application of machine learning, particularly the Bootstrap Forest model, proved valuable in predicting stomatal area size based on a combination of morphological features, genotype, pathogen isolate, and environmental conditions. The machine learning analysis revealed that size-related traits (perimeter, length, and width) had the highest impact on predicting stomatal area size. This is because these traits are directly correlated to area size, providing straightforward information for the models to learn from. When size-related traits were excluded, shape-related traits (LWR, IS & CG, and circularity) became the most important predictors.

Although size-related traits are expected predictors of stomatal area, our machine learning analysis offers deeper insights. First, by systematically evaluating different models, we identified the most effective algorithms for predicting stomatal aperture size in cacao. This knowledge can guide future research on stomatal dynamics in other plant species, optimizing model selection for accurate predictions. Second, our analysis revealed that when size-related traits are excluded, shape-related traits become important predictors. This highlights the subtle interplay between size and shape in determining stomatal aperture and underscores the potential of machine learning to uncover complex relationships among morphological features. This approach can be extended to other plant systems to identify key morphological traits associated with specific physiological functions or responses to environmental stimuli, even when direct measurements are unavailable. Furthermore, the significance of stomatal shape might influence gas exchange efficiency^44^ and/or the ability of pathogens to enter the leaf^6^, deepening the need for further study.

The limited variability of light conditions, pathogen isolates, and genotype within our dataset may explain their minor influence in the machine learning models. This limited variability could have restricted the model’s ability to fully capture the importance of these categorical factors. This study also revealed a minor but differential impact of pathogen isolate and time of treatment on stomatal aperture through machine learning, suggesting that specific pathogen isolates and the duration of exposure potentially influence stomatal dynamics. This approach highlights the potential of machine learning in deciphering complex biological interactions and could be extended to other plant-pathogen systems. Future integration of bioinformatics analyses, such as transcriptomics or metabolomics, could provide deeper insights into the molecular mechanisms and signaling pathways governing these responses. Furthermore, the ability to classify pathogen treatments based on stomatal morphology, as explored with our SVM-RBF model, shows promise for understanding plant-pathogen interactions. Despite the limitations of our current dataset, the finding that stomatal length, circularity, and the distance between IS & CG contribute to classification suggests that variations in stomatal morphology may reflect the type of pathogen interacting with the plant. These traits could potentially be used as markers for resistance in cacao breeding programs, and the image-based approach could be developed into rapid, non-destructive diagnostic tools for early disease detection. This opens up interesting possibilities for developing rapid and non-destructive diagnostic tools for plant diseases. With more robust datasets and further refinement of machine learning models, it could be possible to identify specific pathogens or isolates based on stomatal images, enabling early detection and targeted treatment. This would be particularly valuable in agricultural settings where timely intervention is crucial. Ultimately, a deeper understanding of these plant-pathogen interactions at the stomatal level is critical for developing sustainable disease management strategies and improving cacao yields.

We acknowledge several limitations of the current study. Our experiments were conducted under controlled laboratory conditions with a limited number of cacao genotypes (two), *P. megakarya* isolates (three), and environmental conditions (two light conditions). Furthermore, we used excised leaf segments, which may not fully capture the responses of intact plants. Future studies should address these limitations by increasing the number of biological replicates, expanding the range of genotypes and pathogen isolates (including *P. palmivora* and isolates from diverse geographic regions), investigating stomatal responses under more dynamic and realistic environmental conditions (fluctuating light, temperature, humidity, and CO2 levels), and employing whole-plant assays. Additionally, exploring a wider range of non-pathogenic soil fungi would help determine the specificity of the *R. solani* interaction. Finally, future transcriptomic, proteomic, and metabolomic analyses are needed to elucidate the molecular mechanisms underlying the observed stomatal responses, including identifying specific PAMPs, PRRs, and downstream signaling pathways, as well as examining longer-term impacts on plant health and disease development.

In sum, this study revealed significant differences in stomatal responses between cacao genotypes to *P. megakarya* and *R. solani*, highlighting the complex interplay of genotype, pathogen isolate, and light conditions in regulating stomatal aperture. Crucially, our findings demonstrate the potential for using stomatal morphology, identified through machine learning, as a tool for improving cacao production. The identification of traits like stomatal length and circularity as potential markers for resistance opens up the possibility of marker-assisted selection in cacao breeding programs, enabling the development of varieties with enhanced resistance to BPR. Furthermore, our results pave the way for developing rapid, non-destructive, image-based diagnostic methods for early detection of *Phytophthora* infection, allowing for timely intervention and minimizing yield losses. This work expands our fundamental understanding of plant-pathogen interactions at the stomatal level, while simultaneously providing a foundation for practical applications in cacao breeding and disease diagnostics, crucial advancements for ensuring the long-term viability of this vital crop.

## Materials and methods

### Plant materials

Leaf samples were obtained from clonally propagated cacao trees (genotypes SCA6 and Pound7) at growth stage C. These trees were originally established from semi-hardwood stem cuttings obtained from Penn State between 2006 and 2010 and maintained as bentwood plants to encourage chupon growth. Plants were grown in a controlled greenhouse environment at the USDA-ARS, Beltsville, MD, USA, under tropical conditions (29 –24 °C day/night with 60% relative humidity) with a 12-hour photoperiod (natural daylight supplemented with 400 W HID lighting). Fertigation was performed daily using an automatic irrigation/Dosatron system with Peters’ peat-lite water-soluble fertilizer (18-8-17 + Magnesium and micronutrients). For this study, leaves were randomly collected from distinct individuals of each genotype for triplicate analyses.

### Fungal inoculation and microscopy

Cacao leaves (genotypes SCA6 and Pound7) were harvested and immediately transported to the laboratory with petioles immersed in sterile water. Leaf segments measuring approximately 5 cm x 3 cm were excised using a sterile scalpel. A marker was used to indicate the inoculation site on each abaxial segment. *P. Megakarya* isolates (ZTH0145, GH8, and GH21) were selected to represent geographic diversity within the *P. megakarya* population. Specifically, GH8 and GH21 originated from Ghana, while ZTH0145 was isolated from Cameroon, reflecting the pathogen’s distribution across key cacao-growing regions in West Africa. Agar plugs (1 cm diameter) containing either a control (V8 agar), *P. Megakarya* isolates, or a non-cacao infecting fungus (*Rhizoctonia solani* GC33-C isolate, originally isolated from Bluegrass)^45,46^ were prepared and incubated at 23 °C on V8 agar for 10–13 days to establish fungal cultures. Inoculation was performed by placing an agar plug on the marked area of each leaf segment. Treated leaf segments were maintained in sterile Petri dishes lined with moistened filter paper and sealed with parafilm to prevent desiccation. Leaf segments were subjected to two light conditions: a 12-hour light/12-hour dark cycle (L) (two 30 W Sylvania Cool White fluorescent light tubes, approximately 2,300 lumens per tube, positioned about 45 cm above the Petri dishes) or continuous darkness (D). Microscopic imaging of the inoculation site (where the agar plug remained) was conducted at 0, 24, and 48 hpi using a Nikon ECLIPSE E600 microscope with a Nikon DS-Ri2 camera. All images were stored as JPEG files. The experiment was replicated three times.

### Image analysis

Phenotypic stomata aperture was characterized using SmartGrain software (version 1.3)^47^. This software, primarily designed for seed morphology, was adapted for this study due to its ability to quantify key morphological traits: area, length, width, LWR, perimeter, circularity, and IS & CG^47^. A total of over 7,500 stomata were randomly selected and manually measured per image and time point, with the experiment replicated three times. Stomatal apertures were measured in micrometers (µm) and included in Supplementary Data S1.

### Statistical and bioinformatics analysis

To investigate the intricate interplay of factors influencing stomatal dynamics, we performed a comprehensive statistical and bioinformatics analysis. First, we examined the individual and combined effects of pathogen isolate (including the non-pathogenic *R. solani* isolate GC33-C), cacao genotype (SCA6 and Pound7), and light conditions on stomatal aperture changes over time. ANOVA followed by Tukey’s HSD post-hoc tests were conducted using JMP Pro 17^48^ to identify significant differences among treatment groups (total stomatal samples = 7,558). To further explore the relationships among the measured stomatal traits (area, length, width, length-width ratio, perimeter, circularity, and IS & CG), we calculated Pearson correlation coefficients. Principal component analysis was then employed to visualize the phenotypic diversity and identify key combinations of traits that explain the variation in stomatal responses across treatments. Finally, hierarchical clustering analysis using Ward’s linkage method was performed on all traits to group samples with similar stomatal behavior.

### Machine learning for stomata area size prediction and validation

To further dissect the complex relationships between stomatal morphology, genotype, pathogen isolate, and environmental conditions, we employed machine learning techniques. A dataset comprising 5,904 samples was constructed, ensuring balanced representation across 20 treatment combinations and three-time points (99 samples per condition) to prevent bias. All quantitative stomatal traits (area, length, width, LWR, perimeter, circularity, and IS & CG) were log-transformed using JMP Pro 17. Categorical variables, including light condition (L or D), cacao genotypes (SCA6 and Pound7), and treatments (pathogen isolates or control), were converted into one-hot encoding. The dataset was then divided into training (80%) and testing (20%) sets. JMP Pro’s “Model Screening” function was used to evaluate the performance of nine different machine learning models: Stepwise, Least Squares, Neural Boosted, Generalized Regression Lasso, Boosted Tree, Bootstrap Forest, Decision Tree, SVM (RBF kernel), and k-NN. This model screening was performed with two different approaches. First, we aimed to predict stomatal area size using all available traits, including those directly associated with stomatal area (perimeter, length, and width) and less direct traits (shape-related traits, genotype, treatment, and time). This allowed us to assess the impact of each trait on prediction accuracy. In the second approach, the model screening was conducted again, but this time excluding the three traits directly associated with area size (perimeter, length, and width). This aimed to evaluate the models’ ability to predict stomatal area based on other morphological features, genotypes, pathogen isolates, and environmental conditions. Following the model screening, we employed the Bootstrap Forest model to further analyze the importance of each trait in predicting stomatal area size, both with and without the inclusion of perimeter, length, and width with default setting (Number of trees = 100, number of terms sampled per split = 12, bootstrap rate = 1, and random seed = 0). Similarly, we used the same dataset to assess the ability of machine learning algorithms to classify pathogen isolates (including controls and *Rhizoctonia*). JMP Pro 17’s “Model Screening” function was used to evaluate eight different models: Stepwise, Least Squares, Neural Boosted, Generalized Regression Lasso, Boosted Tree, Bootstrap Forest, Decision Tree, and SVM (RBF kernel), using 10-fold cross-validation.

## Electronic supplementary material

Below is the link to the electronic supplementary material.

Supplementary Material 1

## Data Availability

Phenotypic data are available in the Supplementary file.
